# Genetic diversity and profiles of genes associated with virulence and stress resistance among isolates from the 2010-2013 interagency *Listeria monocytogenes* market basket survey

**DOI:** 10.1371/journal.pone.0231393

**Published:** 2020-04-30

**Authors:** Yi Chen, Yuhuan Chen, Régis Pouillot, Sherri Dennis, Zhihan Xian, John B. Luchansky, Anna C. S. Porto-Fett, James A. Lindsay, Thomas S. Hammack, Marc Allard, Jane M. Van Doren, Eric W. Brown

**Affiliations:** 1 Center for Food Safety and Applied Nutrition, U.S. Food and Drug Administration, College Park, Maryland, United States of America; 2 Consultant, Buenos Aires, Argentina, United States of America; 3 USDA Agricultural Research Service, Wyndmoor, Pennsylvania, United States of America; 4 USDA Agricultural Research Service, Beltsville, Maryland, United States of America; University of Bologna, ITALY

## Abstract

Whole genome sequencing (WGS) was performed on 201 *Listeria monocytogenes* isolates recovered from 102 of 27,389 refrigerated ready-to-eat (RTE) food samples purchased at retail in U.S. FoodNet sites as part of the 2010–2013 interagency *L*. *monocytogenes* Market Basket Survey (*Lm* MBS). Core genome multi-locus sequence typing (cgMLST) and *in-silico* analyses were conducted, and these data were analyzed with metadata for isolates from five food groups: produce, seafood, dairy, meat, and combination foods. Six of 201 isolates, from 3 samples, were subsequently confirmed as *L*. *welshimeri*. Three samples contained one isolate per sample; mmong the 96 samples that contained two isolates per sample, 3 samples each contained two different strains and 93 samples each contained duplicate isolates. After 93 duplicate isolates were removed, the remaining 102 isolates were delineated into 29 clonal complexes (CCs) or singletons based on their sequence type. The five most prevalent CCs were CC155, CC1, CC5, CC87, and CC321. The Shannon’s diversity index for clones per food group ranged from 1.49 for dairy to 2.32 for produce isolates, which were not significantly different in pairwise comparisons. The most common molecular serogroup as determined by *in-silico* analysis was IIa (45.6%), followed by IIb (27.2%), IVb (20.4%), and IIc (4.9%). The proportions of isolates within lineages I, II, and III were 48.0%, 50.0% and 2.0%, respectively. Full-length *inlA* was present in 89.3% of isolates. *Listeria* pathogenicity island 3 (LIPI-3) and LIPI-4 were found in 51% and 30.6% of lineage I isolates, respectively. Stress survival islet 1 (SSI-1) was present in 34.7% of lineage I isolates, 80.4% of lineage II isolates and the 2 lineage III isolates; SSI-2 was present only in the CC121 isolate. Plasmids were found in 48% of isolates, including 24.5% of lineage I isolates and 72.5% of lineage II isolates. Among the plasmid-carrying isolates, 100% contained at least one cadmium resistance cassette and 89.8% contained *bcrABC*, involved in quaternary ammonium compound tolerance. Multiple clusters of isolates from different food samples were identified by cgMLST which, along with available metadata, could aid in the investigation of possible cross-contamination and persistence events.

## Introduction

*Listeria monocytogenes* remain a considerable public health challenge because of their complex ecology and ability to tolerate food-relevant levels of salt and pH and to grow at refrigeration temperatures [1, 2], as well as the severity of invasive listeriosis and high burden of the disease compared to other foodborne pathogens [3, 4]. To better understand the potential public health impact of *L*. *monocytogenes* contamination of ready-to-eat (RTE) foods, many surveys have been conducted to quantify the prevalence and levels of *L*. *monocytogenes* and to generate data for risk assessments. In 2003, the U.S. Food and Drug Administration (FDA) and the U.S. Department of Agriculture (USDA) Food Safety and Inspection Service (FSIS), in consultation with the Centers for Disease Control and Prevention (CDC), published a quantitative assessment of the relative risk to public health from foodborne *L*. *monocytogenes* among 23 selected categories of RTE foods [5]. Multi-faceted efforts were made to better understand risk factors and to assess the risks associated with *L*. *monocytogenes* in RTE foods, including ecology, exposure assessment, dose-response analysis, key events from consuming a contaminated food to infection, and identification of data gaps [2, 5–11]. Parallel efforts were also made to estimate the likelihood and extent of contamination of *L*. *monocytogenes* in higher-volume, higher-risk RTE foods [12–14], and to characterize molecular subtypes for isolates recovered from humans, foods and the food processing environments, and the natural environment [15–21].

Assessing the genetic diversity of *L*. *monocytogenes* is critical to understanding the epidemiology, ecology, and pathogenicity of this pathogen. *L*. *monocytogenes* consists of three major evolutionary lineages, i.e., lineages I, II and III, as well as a rare lineage, that being lineage IV [22]. Each lineage contains strains displaying specific, but slightly overlapping, serotypes, with lineage I containing serotypes 1/2b, 3b, 4b, 4d, 4e and 7, lineage II containing serotypes 1/2a, 3a, 1/2c, and 3c, lineage III containing serotypes 4b, 4a and 4c, and lineage IV containing serotypes 4a and 4c [23]. Four major serogroups as determined by PCR have also been identified that are comprised of nine of the 13 recognized *L*. *monocytogenes* serotypes: IIa (1/2a, and 3a), IIc (1/2c, and 3c), IIb (1/2b, and 3b) and IVb (4b, 4d, nd 4e) [24]. The existence of these genetic lineages and PCR-derived serogroups was further supported by whole genome-based phylogeny comparisons [25]. In contrast, individual serotypes within each PCR serogroup did not always form distinct phylogenetic clades [25].

*L*. *monocytogenes* strains, including some from different countries or continents, can be genetically close and belong to the same clonal complex (CC) or singleton [26, 27]. A CC is defined by a 7-locus multilocus sequence typing (MLST) scheme [22, 27, 28] as a group of strains whose sequence types (STs) differ by no more than one allele from at least one other ST in the group [27, 28]. A ST that differs from all other existing STs in the entire species by at least two alleles defines a singleton [28]. Recently, core genome multilocus sequence typing (cgMLST) analyses showed that CCs identified by the 7-locus MLST scheme generally agree with cgMLST cluster groupings [25, 29]. Isolates within a CC identified by the 7-locus MLST can differ from each other by up to 150 to 170 alleles elsewhere in the genome, depending on the specific cgMLST schemes used [25, 29]. However, isolates in some CCs displayed significantly greater core genome diversity than isolates belonging to other CCs [25]. A new nomenclature, Sub-lineage (SL), was proposed to redefine *L*. *monocytogenes* clones based on core genome variations, which would have classified those CCs with relatively large core genome diversity into multiple clones [29]. Furthermore, analyzing the biodiversity of *L*. *monocytogenes* using CCs, rather than only STs, generates results that are more phylogenetically meaningful because, while CCs may be classified into cgMLST clades, isolates having individual STs do not always form monophyletic cgMLST clades [25, 29, 30].

Comparative characterization of food, environmental, and clinical isolates of *L*. *monocytogenes* from Europe [23, 29–32], North America [19, 33], and Asia [34, 35] revealed predominant genotypes specific to a region or source and the presence of hyper-virulent and stress-resistant genomic features. The relative frequency of occurrence for different clones and ST differed among regions, as well as among isolates from clinical vs. food and environmental sources [19, 29, 30, 33]. These observations and trends in genotype distributions need to be further investigated with additional isolates, preferably with well-characterized metadata.

Following the 2003 FDA-FSIS risk assessment and several other product-specific risk assessments to evaluate the impact of interventions [5, 6, 36], FDA and FSIS issued regulations and guidance on risk-based *Listeria* control [1, 37–41]. The food industry, working with academia, also developed guidance for risk-based *Listeria* control and made changes to industry practices [42–46]. To assess potential changes in *L*. *monocytogenes* contamination of RTE foods following the implementation of these guidance and control measures in the early and mid-2000’s, a multiyear, interagency *L*. *monocytogenes* Market Basket Survey (*Lm* MBS) was conducted between 2010 and 2013 by FDA, USDA FSIS, and the USDA Agricultural Research Service (ARS) to estimate the prevalence, levels, and types of *L*. *monocytogenes* in selected RTE foods purchased at retail across four FoodNet sites in the U.S. [13]. Using a surveillance sampling plan stratified according to food consumption, geographic location, retail store type, and other factors, a total of 27,389 RTE food samples comprising 16 product categories were collected and tested; the proportion of positive samples ranged from zero to 1.07% [13]. These results showed a significant decrease (*p*<0.001) in the prevalence of *L*. *monocytogenes* in RTE foods such as deli salads without meats, deli meats, smoked seafood, seafood salads, and soft-ripened and semisoft cheese compared to a large-scale 2000–2001 market basket survey [12, 13] and other comparable surveys in the U.S. a decade ago that reported positive rates of 1.0 to 6.4% [47, 48].

The objectives of the present study were to: 1) assess the biodiversity and relatedness of the *L*. *monocytogenes* isolates retained from the *Lm* MBS for which extensive metadata were available, with regard to food groups, sources and packaging locations, and 2) identify key genomic features contributing to virulence potential in the host, along with stress response and persistence in food or environment.

## Materials and methods

### Description of *L*. *monocytogenes* food isolates

A total of 201 culture-confirmed and viable isolates of *L*. *monocytogenes* were recovered from 102 of the 27,389 refrigerated RTE food samples analyzed during the *Lm* MBS [13]. The food samples were collected on a weekly basis between 2010 and 2013 at retail stores across four U.S. FoodNet sites (i.e., California, Maryland, Connecticut, and Georgia). The 201 isolates were recovered from five of six broad food groups analyzed comprising 16 food categories: Seafood (n = 12 samples), Produce (n = 30 samples), Dairy (n = 8 samples), Meat (n = 16 samples), and Combination Foods (n = 36 samples). A sixth food group, Eggs (including hard cooked eggs and deviled eggs), was also sampled in the survey, but no viable *L*. *monocytogenes* were found in these samples (total 456 samples tested) [13]. The food category and the number of positive samples for each food group included: i) smoked seafood (n = 3), and ii) seafood salad (n = 9) for the Seafood group; i) raw cut vegetables (n = 18), ii) low-acid cut fruits (n = 9), and iii) sprouts (n = 3) for the Produce group; i) artisanal cheese (n = 4), ii) cultured milk product (n = 1), and iii) raw milk (n = 3) for the Dairy group; i) deli meat (n = 15), and ii) sausage (n = 1) for the Meat group; and i) deli-type salad without meat (n = 21), ii) deli-type salad with meat (n = 4), and iii) sandwiches (n = 11) for the Combination Foods group. For sprouts, only one isolate was obtained from each of the three positive samples [49]. For the remaining 99 positive food samples, two isolates were retained from each positive sample. Each isolate was recovered from a food sample with known manufacturing location, that is, the food sample was prepackaged in a processing facility or was made and/or deli-packaged in store. These 201 isolates are hereafter referred to as the 2010–2013 *Lm* MBS collection.

### Whole genome sequencing analyses

All isolates were sequenced on an Illumina MiSeq platform (250-bp, paired-end reads, Illumina, Inc., San Diego, CA) using the Nextera XT Library Preparation Kit per the manufacturer’s instructions [50]. The genomic sequence contigs for each isolate were *de novo* assembled using Qiagen CLC Genomics Workbench 11.1 (Aarhus, Denmark). We analyzed these genomes by a previously developed cgMLST typing scheme utilizing the cgMLST tool built in Ridom^©^ SeqSphere^+^ (Ridom^©^ GmbH, Münster, Germany) targeting 1,827 core genes of *L*. *monocytogenes* [25]. The percentage of “good” *L*. *monocytogenes* cgMLST targets of each genome was determined. A good cgMLST target means that a gene in an isolate aligns with a reference gene in the cgMLST scheme by 80% identity, has the same length as the reference gene ± three triplets, and has no ambiguities or frame shifts compared to the reference gene. A percentage of >95% good targets for a genome means that that genome contains >95% of the pre-defined 1,827 core genes, indicating suitable applicability of the genome assemblies and the cgMLST scheme [51]. A neighbor-joining tree was constructed using pairwise allelic differences. The combination of clustering in the neighbor-joining tree and the number of allelic differences was used to determine whether two isolates from the same sample were likely the same strain. When CLC assemblies had questionable quality which may affect the determination of isolate relationship, we assembled the genome(s) by SPAdes [52] for cgMLST analyses. We combined WGS with available metadata for final analysis of these clusters.

### Determination of clonal complex, PCR-serogroup, lineage and premature stop codon (PMSC) in *inlA*

The *in-silico* MLST implemented in the SeqSphere^+^ software was used to determine the sequence type of these isolates. Clonal complexes and singletons were then assigned based on the definition by Ragon et al. [28] and profiles curated in the Institut Pasteur MLST *Listeria* database (http://bigsdb.pasteur.fr/Listeria/Listeria.html). *In-silico* PCR-serogroup identification was performed by determining the presence of targets used to define four major PCR-serogroups: i) IIa, which was shown to correspond to serotypes 1/2a and 3a by anti-serum based serotyping; ii) IIc, corresponding to serotypes 1/2c and 3c; iii) IVb, corresponding to serotypes 4b, 4d and 4e, and iv) IIb, corresponding to serotypes 1/2b and 3b [24]. Each isolate was determined to be lineage I or lineage II using both serogroup information and cgMLST phylogeny. The cgMLST phylogeny was also used to identify lineage III strains from the *Lm* MBS. None of the 201 isolates in our collection belonged to lineage IV. The *inlA* sequences were extracted from the whole genome sequences using CLC Genomics Workbench and aligned using MEGA 7.0 [53]. Internalin A (InlA), encoded by *inlA*, is a major factor facilitating *L*. *monocytogenes* crossing human intestinal barrier during infection; PMSCs in *inlA* lead to a truncated inlA, which resulted in attenuated virulence of *L*. *monocytogenes*. PMSCs were determined manually using *inlA* sequences.

### Determination of the presence of virulence-associated genes, genes involved in stress resistance, and plasmids

Nucleotide BLAST was performed to determine the presence of previously surveyed genes associated with virulence and stress response [29, 32] among isolates genetically confirmed as *L*. *monocytogenes* by WGS analyses. A threshold of ≥70% query coverage with ≥80% sequence identity of BLAST alignment indicated the presence of a gene or genomic island [54]. The contigs of each shotgun genome were used as query genomes and compared by BLAST with complete sequences of 52 *Listeria* plasmids deposited in the GenBank as of November 10, 2019 (S1 Table) and the *repA* gene (NCBI Accession: CP006595.1, from the plasmid of *L*. *monocytogenes* strain R2-502) was compared by BLAST with all shotgun genomes. We considered a contig as a plasmid contig if the query coverage was ≥60% and sequence identity was ≥70%. We considered *repA* to be present in a shotgun genome if the BLAST had query coverage of ≥60% and sequence identity of ≥70%. Because 90% of the plasmids published in NCBI are ≥10 Kbp, and some regions in plasmids and chromosomes could be homologous [55], we determined that a shotgun genome contained a plasmid if the following two criteria were met. First, in the shotgun genome, the total length of contigs determined to be plasmid contigs exceeded 10 Kbp; second, *repA* is present in one of the plasmid contigs. If the total length of plasmid contigs in a shotgun genome were less than 10 Kbp, we considered the determination of plasmid presence inconclusive for that shotgun genome.

### Statistical analyses of the biodiversity of *L*. *monocytogenes*

For statistical analysis, we removed duplicate isolates. For this study we note that duplicate isolates were defined as follows: if two isolates from the same food sample belonged to the same cluster and thus likely were the same strain as determined by the 1827-gene cgMLST, we considered them as duplicative and included only one for further analysis. If isolates from different food samples belonged to the same cluster, we did not consider them as duplicates regardless of their allelic differences, in order to capture the difference in the prevalence of each genotype. We recognize however, it could be argued that this definition has concerns in that if strains from two different food samples cluster together and share the same sequence type then these strains could also be considered “duplicates” for other types of analysis. The metadata collected in the *Lm* MBS [13] were also used to delineate the isolates by CC, serogroup and lineage, as well as by food group, food category, and product packaging location. The binomial exact test was used to evaluate if the proportions of isolates belonging to lineage I and lineage II differed significantly in this study. Fisher’s exact test was used to evaluate if the proportions of isolates belonging to lineage I and lineage II, and the proportions of isolates with and without an *inlA* PMSC, differed significantly between the present study and results reported in previous subtyping studies on a 2000–2001 isolate collection (from a previous survey with comparable study design that yielded 502 isolates from 31,705 RTE samples) [12, 20]. Genetic diversity, as determined by the number of CCs and/or singletons per food group or manufacturing location, was evaluated by using Shannon’s index [56], where differences between food groups and manufacturing locations were tested using a bootstrap method [57]. More specifically, Shannon’s index was evaluated on 10,000 random samples generated by computer simulation with the marginal values that equaled to the observed ones. When comparing between food groups, a Bonferroni correction was made because of multiple statistical tests. The statistical analysis was implemented using R [58].

## Results and discussion

The isolate collection retained from the *Lm* MBS offered a novel opportunity for WGS analyses to determine the temporal and spatial relatedness of *L*. *monocytogenes* strains across high volume, high risk RTE foods. The *Lm* MBS was a large-scale multi-year survey that analyzed 27,389 food samples across 16 food categories across six broad food groups: seafood, produce, dairy, meat, eggs, and combination foods. The genomes were deposited at GenomeTrakr database housed at National Center for Biotechnology Information (NCBI) and their Biosample numbers and Sequence Read Archive IDs are listed in S2 Table.

### Phylogenetic analysis of *L*. *monocytogenes* isolates

From the 102 food samples tested positive for *L*. *monocytogenes* from the *Lm* MBS, we retained 2 isolates from each of 99 non-sprout samples and a single isolate from each of the remaining 3 sprout samples for more detailed characterization. All 201 isolates were subjected to WGS sequencing. Six isolates (from two egg salad samples and one egg salad sandwich sample) that had been determined by biochemical characterization and phenotypic-based methods to be *L*. *monocytogenes* [13] were confirmed as *L*. *welshimeri* using WGS in this study; these six isolates from three non-sprout samples were excluded from further analyses. Of the remaining 195 isolates, 193 isolates contained ≥99.0% and two isolates contained ≥98.5% of “good” *L*. *monocytogenes* cgMLST targets (S2 Tabe).

**Fig 1:**
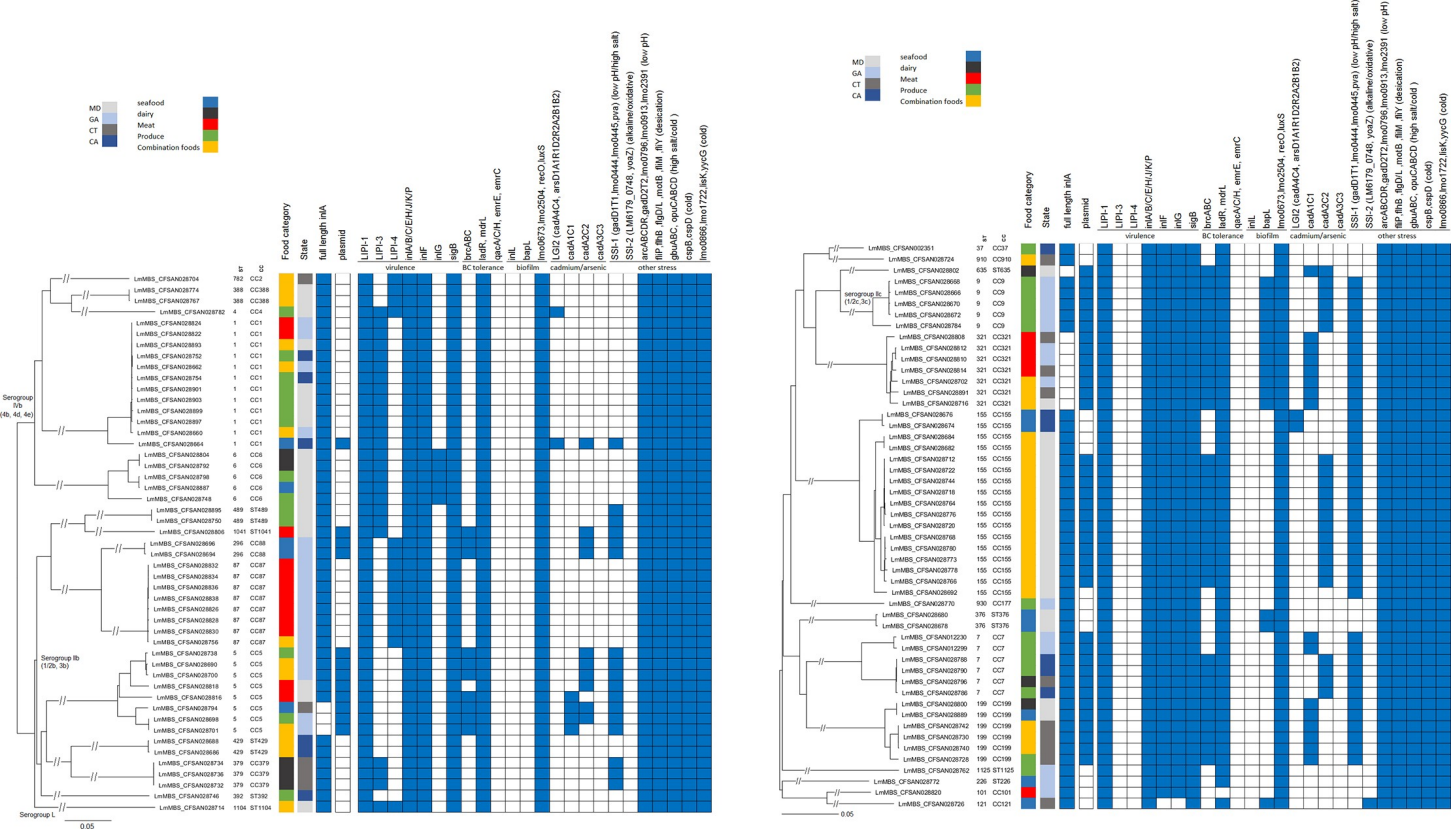
Phylogenetic trees of (A) lineage I (n = 49), and (B) lineage II (n = 51) *L*. *monocytogenes* isolates from five food groups included in the 2010–2013 *Lm* MBS, constructed by the neighbor-joining algorithm using pairwise allelic differences generated by core genome multilocus sequence typing (cgMLST). Shown is the relatedness of the isolates and, for each isolate, the designation of serogroups, clonal complex (CC), sequence type (ST), the presence (filled space) and absence (open space) of full length inlA, plasmid, and selected genes associated with virulence and stress resistance. The food categories are color coded.

For 3 of the 96 non-sprout samples that tested positive for *L*. *monocytogenes*, both isolates from each sample (i.e., CFSAN028701/CFSAN028702, CFSAN028767/CFSAN028768, and CFSAN028773/CFSAN028774) belonged to different MLST-defined CCs and differed by more than 160 cgMLST alleles; therefore, we considered both isolates as different strains and included both for further analysis. For the 92 of the remaining 93 non-sprout samples, the two isolates retained from each sample differed by ≤9 alleles; therefore, only one isolate from each of these 92 samples was included for more detailed analysis. For the remaining non-sprout sample, both isolates (i.e., CFSAN028677 and CFSAN028678) differed by 13 alleles, so we used an alternative method, SPAdes, to assemble these genomes [52]. We found that these isolates differed by 5 alleles if SPAdes assembly of CFSAN028677 (N50, 303384 nt), which had higher quality than the CLC assembly (N50, 151631 nt) (S2 Table), was used for cgMLST analysis. Different assembly methods for CFSAN028678 did not affect the results. Thus, the 13 allelic differences initially calculated could be due to low quality assembly of CFSAN028677 by CLC and we included only a single isolate in this sample for further analysis. After removing 93 duplicates, a total of 102 isolates were identified from the *Lm* MBS isolate collection. The phylogenetic trees for lineage I and lineage II isolates are shown in Fig 1. The multi-clone contamination of a single sample illustrated the value of retaining more than one isolate per sample for WGS analysis to accurately assess genotype diversity.

### Diversity of serogroups and lineages among the *L*. *monocytogenes* isolates

**Fig 2:**
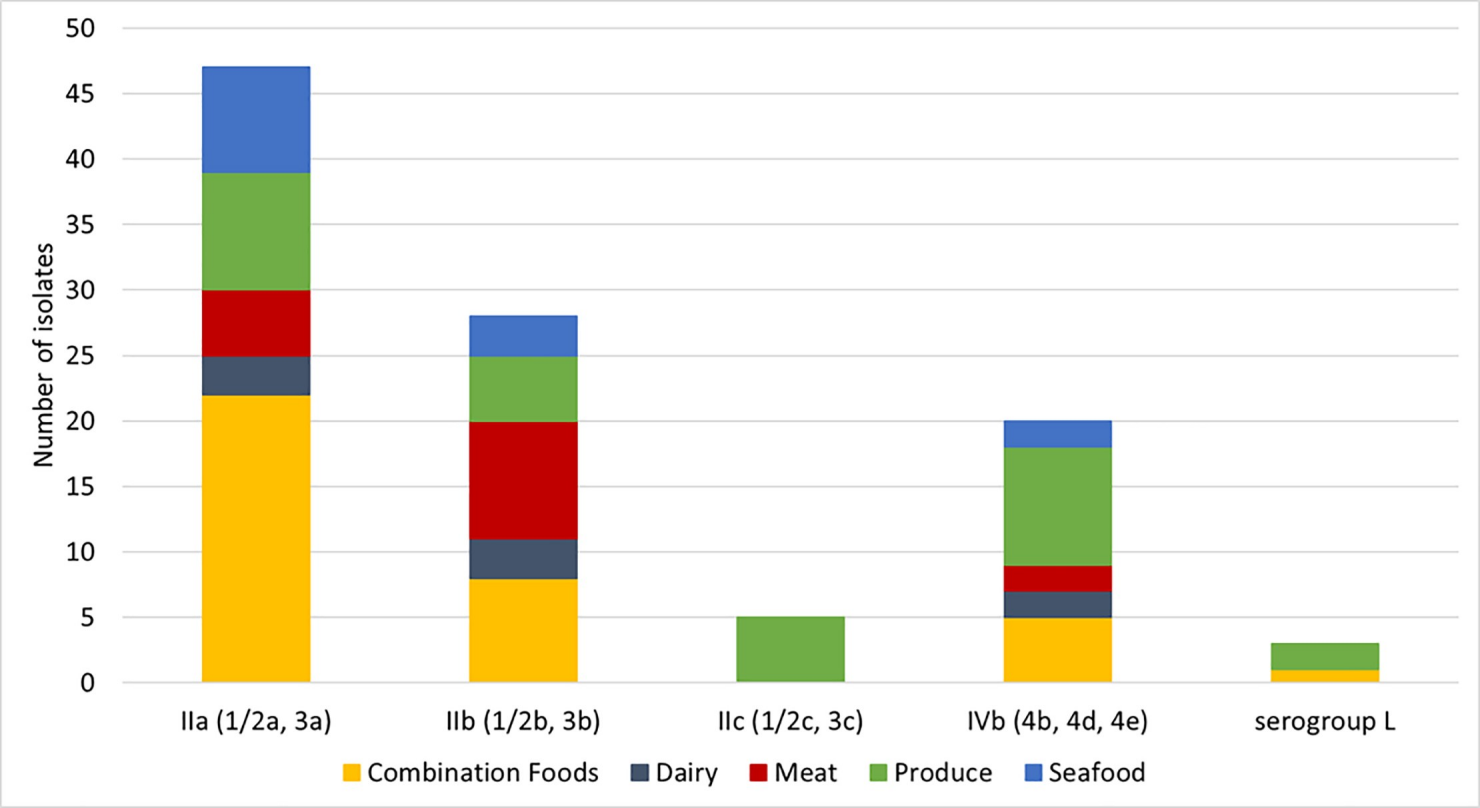
Prevalence of serogroups IIa, IIb, IIc, IVb, and L among *L*. *monocytogenes* isolates (n = 102) obtained from five food groups. Serogroup L does not have the markers of serogroups IIa, IIb, IIc and IVb. WGS determined that one serogroup L isolate belonged to lineage I and the other two serogroup L isolates belonged to lineage III.

Among the 102 isolates from the *Lm* MBS that were genetically confirmed as *L*. *monocytogenes* by WGS analyses, there were 49 lineage I isolates, 51 lineage II isolates, and 2 lineage III isolates. The most frequently identified serogroup was IIa (1/2a and 3a, n = 46), followed by IIb (1/2b and 3b, n = 28), IVb (4b, 4d, 4e, n = 20), IIc (1/2c and 3c, n = 5), and serogroup L without any serogroup markers in the genome (n = 3). One serogroup L isolate, belonging to singleton ST1104, was subsequently confirmed as lineage I (Fig 1) and the remaining two serogroup L isolates were confirmed as lineage III (S1 Fig) via WGS. Fig 2 shows the diversity of serogroups within the five food groups from which *L*. *monocytogenes* isolates were obtained. The isolates belonging to serogroup IIa, IIb, or IVb were obtained from combination foods, dairy, meat, produce and seafood samples (Fig 2). All five isolates in the serogroup IIc were obtained from produce samples. Table 1 shows the proportion of lineage I (48.0%) and that of lineage II (50.0%); these were not statistically different (p>0.05). Isolates among lineage I differed by up to 1296 alleles, isolates among lineage II differed by up to 1478 alleles, and the two lineage III isolates differed by 1 allele. Within lineage I, isolates of serogroup IVb differed by up to 1190 alleles and isolates of serogroup IIb differed by up to 1272 alleles.

**Table 1:** Proportion of *L*. *monocytogenes* isolates obtained from large-scale market basket surveys: Present study compared with previous study^a^.

Molecular subgroup	This study, 2010–2013 survey isolate collection			Previous studies, 2000–2001 survey isolate collection			*P* (Fisher’s test) ^b^
	No. isolates for subgroup	Total No.	Percent	No. isolates for subgroup	Total No.	Percent	
Lineage I	49	102	48.0%	187	502	37.3%	< 0.05
Lineage II	51		50.0%	313		62.4%	
Lineage III	2		2.0%	2		0.4%	
*inlA* without PMSC	91	102	89.2%	275	502	54.8%	< 0.001
*inlA* with PMSC	11		10.8%	227		45.2%	

^a^ For the present study, isolates were obtained from the 2010–2013 *Lm* MBS [13] and the molecular subgroup was determined based on WGS data. Isolates obtained from a previous 2000–2001 survey study [12] were further analyzed and molecular subgroups were determined for lineages as reported by Gray et al. [16] and for *inlA* premature stop codon mutations as reported by Van Stelten et al. [20] with update by Chen et al. [83].

^b^ The *p* value shown is for the comparison between isolates from the 2010–2013 and the 2000–2001 surveys.

### Diversity of clonal complexes and sequence types among the *L*. *monocytogenes* isolates

**Fig 3:**
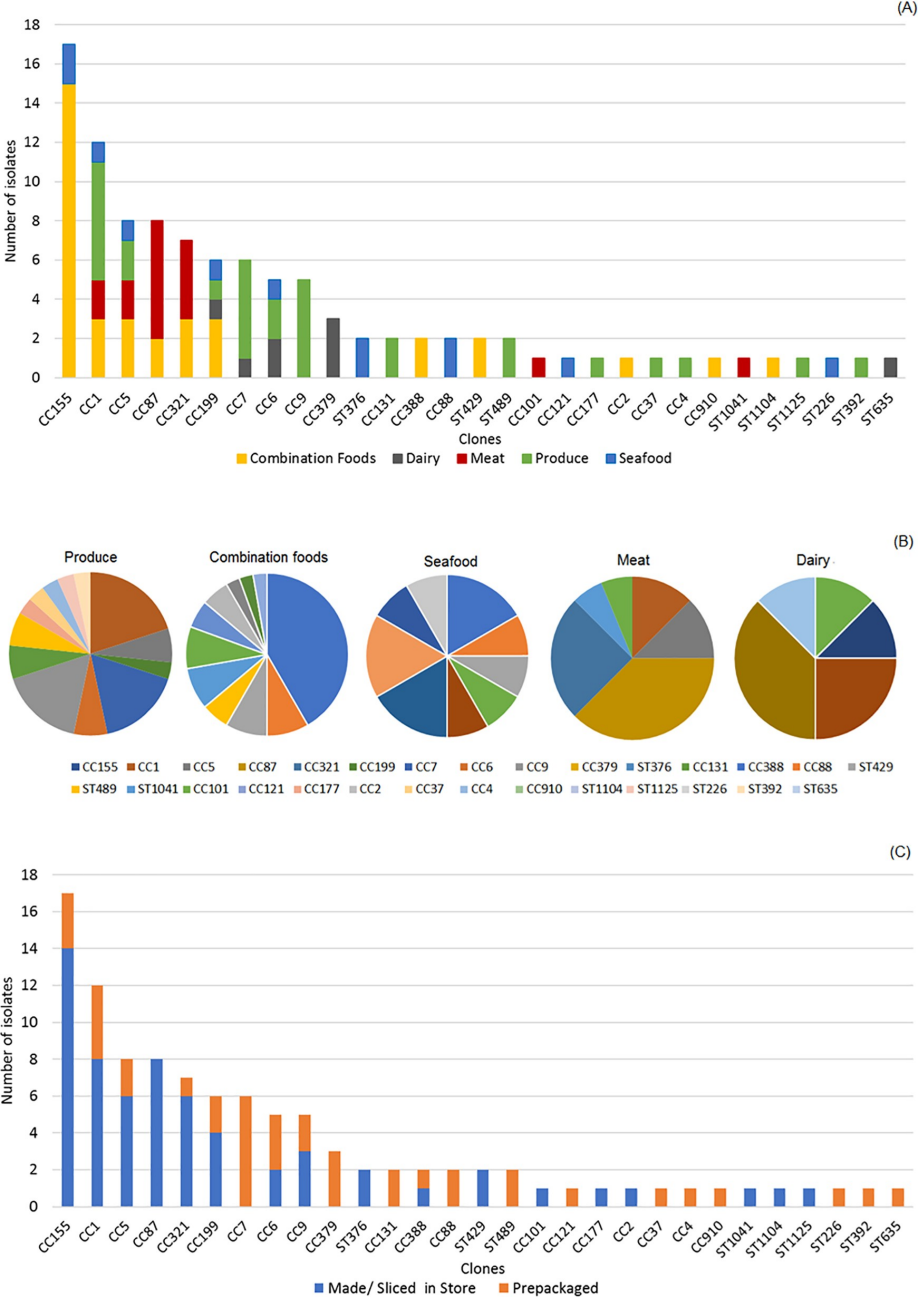
Diversity in clonal complex (CC) and sequence type (ST) of *L*. *monocytogenes* isolates from the 2010–2013 *Lm* MBS survey, by (A) and (B) food group, and (C) manufacturing location of food samples in which isolates were obtained. Fig 3A illustrates the number of isolates in each food group in each clone. Fig 3B illustrates the percentage of each clone in each food group.

Among the 102 isolates of *L*. *monocytogenes* from the *Lm* MBS, *in-silico* MLST analysis based on WGS identified a total of 29 STs, belonging to 20 CCs and 9 singletons. Furthermore, the maximum pairwise allelic differences in each of the clones was ≤134; thus, using cgMLST diversity would have defined the same clones as those by MLST because a survey of over 50 *L*. *monocytogenes* clones using this cgMLST method established that isolates belonging to a typical MLST-defined clone differed by up to 167 cgMLST alleles [25]. The number of CCs/singletons varied (but is not significantly different) among the five food groups (Fig 3A and 3B). The relative abundance of each clone (i.e., the number of isolates belonging to a distinct CC or singleton) also varied. Isolates from produce, combination foods, and seafood groups harbored 13, 11 and 9 clones, respectively (Fig 3B). By comparison, meat and dairy isolates had 6 and 5 clones, respectively (Fig 3B). The Shannon’s diversity index, which evaluates both the number of clones and their frequency of occurrence, varies among the food groups (Table 2, Fig 3B), with isolates from produce displaying the greatest diversity. Pairwise comparisons of the isolates per food group using Shannon’s index with Bonferroni correction (to account for multiple tests) showed that diversity did not differ significantly (p>0.005) between any two food groups (Table 2). The food samples in the *Lm* MBS [13] were either a prepackaged food made in a manufacturing facility or a food made or sliced or packaged in a retail store. Among the 102 isolates from the *Lm* MBS, 40 isolates were recovered from prepackaged foods and the remaining 62 were from foods made/sliced in stores (i.e., deli-packaged). Regarding packaging, a total of 20 and 17 clones were identified from prepackaged and deli-packaged food samples, respectively (Fig 3C). The Shannon’s index based on the product manufacturing and packaging location was 2.83 for isolates from prepackaged foods, and 2.43 for deli-packaged foods, which were not significantly different (p = 0.11). Related to geography, isolates obtained from samples collected in Maryland, Connecticut, Georgia, and California contained 42, 14, 34 and 12 clones, respectively. Maryland had 15 isolates of CC155, California had 2 isolates of CC155, and the other two states did not have any CC155, which indicated that CC155 may be strongly associated with the foods in Maryland. Georgia had 8 isolates of CC87 and 5 isolates of CC9, and other states did not have these clones. For clones other than CC155, CC87 and CC9, the number of isolates per clone and per state is not large enough to determine any potential associations between or among clones and states. The clonal diversity, by food category, among the 102 isolates recovered from the *Lm* MBS is provided in S2 Fig.

**Table 2:** Pairwise comparisons of isolates per food group in the diversity of clonal complexes^a^.

Food Group: Shannon’s index	Produce	Seafood	Combination foods	Meat	Dairy
Produce: 2.32		p = 0.90	p = 0.20	p = 0.03	p = 0.22
Seafood: 2.14	p = 0.90		p = 0.83	p = 0.08	p = 0.05
Combination foods: 1.98	p = 0.20	p = 0.83		p = 0.27	p = 0.69
Meat: 1.58	p = 0.03	p = 0.08	p = 0.27		p = 0.87
Dairy: 1.49	p = 0.22	p = 0.05	p = 0.69	p = 0.87	

^a^ Diversity of clonal complexes within a food group is represented by Shannon’s index. Pairwise comparison between two food groups is made by using Shannon’s index with Bonferroni correction, where p≤0.005 indicates a significant difference. No significant differences were found.

The most abundant clone in this study was CC155 of serogroup IIa (17 of 102 isolates, or 16.7%), followed by CC1 of serogroup IVb (12 of 102 isolates, or 11.8%), CC5 of serogroup IIb (8 of 102 isolates), CC87 of serogroup IIb (8 of 102 isolates), and CC321 of serogroup IIa (7 of 102 isolates). These top 5 CCs accounted for 51% of the 102 isolates from the *Lm* MBS. In addition to the CC1 isolates, regarding hypervirulent clones [30], we found 1 isolate of CC2, 1 isolate of CC4, and 5 isolates of CC6 (Fig 3). These clones were the etiological agents for multiple listeriosis outbreaks [25].

The prevalence, pathogenicity, and persistence of *L*. *monocytogenes* clones within and among collections of isolates have been the subject of many previous studies. Nielsen et al. [23] reported that the 5 most prevalent clones, in descending order of prevalence, were CC121, CC9, CC8, CC155, and CC6 among 353 RTE food isolates from an EU-wide baseline survey conducted in 2010–2012. Maury et al. [30] analyzed 4049 isolates from various food categories and sources collected in France and the top 5 most prevalent clones, in descending order of prevalence, were CC121, CC9, CC1, CC2, and CC8+CC16. In other studies, the top 5 clones were CC5, CC1, CC224, CC363, and ST191 among a collection of 121 isolates from milk and milk processing equipment in the U.S. [59]; CC1, CC2, CC6, CC554, and CC315 among 77 serotype 4b isolates from various food categories and food processing environments between 1985 and 2011 in U.S. [33]; CC3, CC204, CC155, CC1, and CC9 among isolates from various food categories collected between 1931 and 2015 in Australia [60]; CC9, CC121, CC2, CC204 and CC5 among 142 isolates from various food categories collected between 2011 and 2014 in Switzerland [61]; CC121, CC1/CC2/CC9 (i.e., clones of equal prevalence separated by “/”) and CC321/CC155/CC11/CC288 among 60 isolates from retail meat collected between 2004 and 2007 in Japan [34]; and CC8, CC1, CC87, CC155, and CC9/CC3 among 80 isolates from retail RTE foods collected between 2012 and 2014 in China [35]. Comparison of results from the present study and the above mentioned seven studies showed that CC1, a “hypervirulent clone” [30] with the most recent common ancestor estimated to be around 1876 [29], is a common clone among food isolates from many countries. However, it is not among the top 10 most prevalent clones in several of the studies discussed above; for example, CC1 comprised 1.7% of the RTE food isolates in Europe [23], compared to 11.8% of isolates from the *Lm* MBS. In the studies discussed above, CC155, CC9 and CC121 were frequently isolated from foods. Specifically, CC155 comprised 16.7% and 10.3% of isolates from the *Lm* MBS and the Australia collection [60], respectively, but only comprised 1.6% of all isolates in France [30]. The most abundant clone among the collections of isolates in France, Switzerland and Japan [30, 34, 61] was CC121, which was found in 26.8%, 13.6% and 18.6% of each collection, respectively. In contrast, CC121 was identified in only 1% of the 102 isolates from the *Lm* MBS. The most prevalent clone in Australia (CC3; 35.3% among food isolates) was not found among the *Lm* MBS isolates. Of note, these comparisons were made based on CCs either directly available in the previous studies or converted from STs when CCs were not reported.

These differences in findings from this and the published studies may reflect, in part, regional differences in *L*. *monocytogenes* biodiversity. Such differences may also be explained, in part, by differences in the nature, numbers, and types of products selected for evaluation in the various studies, the recovery methods and culture media used, and/or the different purposes and sampling designs (e.g., market basket survey vs. targeted surveillance vs. outbreak investigations). For example, the European study [23] included isolates mostly from smoked fish, heat-treated meats, and soft and semisoft cheese, along with five isolates from produce. For the *Lm* MBS, a substantial proportion of the isolates were from combination foods (deli-type salad and sandwiches) and from produce (Fig 3), consisting of 34% and 29% of the isolates, respectively. As another example, the isolates in France were from multiple sampling efforts with different purposes, including those associated with food alerts, investigations following listeriosis detection, industry sampling, and food surveillance activities [30]. By comparison, the isolates for the *Lm* MBS were collected in a baseline survey with a defined sampling plan that was stratified according to consumption, geographic location, the type of retail stores and other factors [13].

### Comparison of the proportions of isolates by lineage for isolates from other studies of similar scope and magnitude

The *L*. *monocytogenes* isolates from the 2000–2001 market basket survey reported by Gombas et al. [12] were extensively characterized to determine their serotypes and pulsotypes [18], ribotypes, *hly* profiles and lineages [16], and the presence or absence of 18 PMSCs in *inlA* [20]. Compared to the isolates collected from RTE foods during the 2000–2001 survey (Table 1), the proportion of lineage I isolates increased from 37.3% to 48.0% in the 2010–2013 *Lm* MBS and the proportion of lineage II isolates decreased from 62.4% to 50.0% (Table 1). The 2000–2001 survey and the 2010–2013 *Lm* MBS survey have comparable study design, and thus, allow for meaningful statistical comparison. These changes in the proportions of lineage I and lineage II between the isolates from the 2000–2001 survey and the isolates from the 2010–2013 *Lm* MBS collection were statistically significant (p<0.05).

Although several studies reported that lineage II isolates were significantly overrepresented in foods [61–67], other studies reported that lineage I isolates predominate in foods. For example, Sauders et al. [68] reported that lineage I isolates represented 55.8% of a collection of 151 environmental isolates and 5 food isolates of *L*. *monocytogenes* from 121 retail establishments inspected between 2005 and 2006 in New York State. As another example, lineage I isolates represented 82.6% of a collection of 33 isolates from imported seafood in China between 2007 and 2008 [69], 48.5% of a collection of 136 food isolates obtained between 1931 and 2015 in Australia [60], 46.5% of 60 food isolates collected from retail meat in Japan between 2004 and 2007 [34], 48.8% of a collection of 80 isolates recovered from various retail RTE food isolates in 24 cities in China between 2012 and 2014 [35], 60% of a collection of 81 isolates from RTE meat products in processing plants and retail establishments in central Portugal sampled between 2011 and 2013 [70], and 82.8% of a collection of 64 food isolates from retail establishments and supermarkets in Singapore sampled between 2011 and 2012 [71].

### Presence of PMSCs in *inlA* and comparison with other studies

Regarding PMSCs in *inlA*, 11 isolates, representing 10.8% of the 102 isolates from the *Lm* MBS collection, harbored truncated *inlA* genes; this was significantly lower than the proportion (89.2%) of isolates without a PMSC (p<0.001). All seven CC321 isolates contained PMSC mutation type 3 [72]; in addition, three CC5 isolates contained PMSC mutation type 1 [72] and formed a monophyletic clade (Fig 1); finally, one of the 11 isolates belonged to a singleton, ST635, and contained mutation type 7 [73]. The comparable survey scale and sampling design in the 2000–2001 and the 2010–2013 U.S. surveys allowed us to perform meaningful statistical comparisons on the isolate collections from these two surveys. The percentage of isolates containing *inlA* PMSCs significantly decreased (p<0.001) from isolates collected in the 2000–2001 survey (45.2%) to the 2010–2013 survey (10.8%).

Isolates belonging to lineage I or those with a full-length inlA are generally associated with a greater virulence potential than those belonging to lineage II or those with *inlA* PMSCs [66, 74–76]. The present study found a significant shift toward a higher proportion of lineage I isolates and a higher proportion of isolates with intact *inlA* among food isolates compared with isolates characterized by Gombas and colleagues [14] (Table 1). This finding may suggest a shift toward a higher average virulence potential in *L*. *monocytogenes* in selected RTE food groups in the U.S. Because our analysis was based on the comparable isolate collections available and thus limited to the timeframes and RTE foods included in the two comparable surveys in several U.S. FoodNet sites [12, 13], further study is needed to substantiate this finding. In vitro or in vivo assays are also needed to confirm the shift of the virulence potential for a representative strain from the *Lm* MBS and other food, clinical, and environmental isolates.

Previous studies showed that *inlA* PMSCs occurred more frequently in lineage II isolates than in lineage I isolates [66, 72] and that isolates with *inlA* PMSCs were overrepresented in food and environmental samples, but underrepresented in clinical samples [20, 77, 78]. In the present study, the percentage of lineage II isolates containing *inlA* PMSCs was 15.7%. In two other studies, the percentages of *inlA* PMSCs were even lower. Gorski et al. [15] reported that 2.7% of the 112 *L*. *monocytogenes* isolates from naturally contaminated watersheds contained *inlA* PMSCs, and Wang et al. [21] found that 2.4% of 422 isolates from the retail deli environments harbored truncated *inlA*. Therefore, analyses of additional strain collections should be performed to determine the overall prevalence of *inlA* PMSCs in food, environmental and clinical isolates.

### Presence of plasmids and select virulence genes and genes implicated in persistence

The presence of major virulence genes and genes implicated in stress response and environmental persistence [29, 32] were determined for the *Lm* MBS isolates (Fig 1). *Listeria* Pathogenicity Island 1 (LIPI-1, containing *prfA*, *plcA*, *hly*, *mpl*, *actA* and *plcB*), the first-identified pathogenicity island for *L*. *monocytogenes*, was present in all the isolates. LIPI-3 (*llsAGHXBYDP*), encoding a listeriolysin S, was found only in lineage I isolates (25 out of 49, 51%). LIPI-4 (genes LM9005581_70009 to LM9005581_70014), implicated in neural and placental infections, was found only in lineage I isolates (15 out of 49, 30.6%), belonging to CC2, CC388, CC4, CC88, CC87 and singleton ST1104. Furthermore, all isolates from the *Lm* MBS contained sigma factor B (*sigB*), a stress response and virulence regulator, and major internalins, including *inlABCEGHJK*. The stress survival islet 1 (SSI-1), involved in tolerance of low pH and high salt concentrations, was present in 17 of 49 (34.7%) lineage I isolates and 41 of 51 (80.4%) lineage II isolates between which the difference was significant (p<0.05). Stress survival islet 2 (SSI-2), involved in tolerance of alkaline and oxidative stress, was present only in the CC121 isolate. The plasmid-borne benzalkonium chloride (BC) resistance cassette (*bcrABC*) was present in 10 of 49 (20.4%) lineage I isolates, and 35 of 51 (68.6%) lineage II isolates; this difference was significant (p<0.05). Other plasmid-borne BC tolerance genes, *qacA*, *qacC*, *emrE* and *emrC*, or the transposon-borne BC tolerance gene, *qacH*, were not found in any of the isolates. The chromosome-borne BC resistance genes *ladR* and *mdrL* were found in all isolates. *Listeria* genomic island 2 (LGI2), involved in cadmium and arsenic resistance, was found in isolates belonging to CC4, CC1, and CC155; cadmium resistance gene cassette *cadA3C3* was not found in any isolates. The plasmid-borne cadmium resistance gene cassettes, *cadA1C1* and/or *cadA2C2*, were found in 12 out of 49 lineage I isolates and 37 out of 51 lineage II isolates; this difference was significant (p<0.05). Genes involved in tolerance of low pH, desiccation, high salt concentration and cold conditions, such as *arcABCDR*, *gadD2T2*, *lmo0796*, *lmo0913*, *lmo2391*, *fliP/M/Y*, *flhB*, *flgD/L*, *motB*, *gbuABC*, *opuCABCD*, *cspB/D*, *lmo0866*, *lmo1722*, *lisK* and *yycG*, were found in all isolates. The *inlL* gene, involved in biofilm formation was not found in any of the isolates. Likewise, the *bapL* gene, involved in biofilm formation, was not found in lineage I isolates, but found in 15 out of 52 (29.4%) of lineage II isolates, belonging to CC9, CC321, singleton ST376 and CC121. Other genes involved in biofilm formation, such as *lmo0673*, *lmo2504*, *recO*, and *luxS* were found in all isolates. The two lineage III isolates contained SSI-1, *arcA/R*, and did not contain LIPI-3, LIPI-4, LGI2, SSI-2, or *arcB/C/D*.

By BLAST comparison of all shotgun genomes against publicly available plasmid sequences, we found plasmid contigs in 49 of the 102 deduplicated isolates, which included 12 lineage I isolates (24.5% of the 49 lineage I isolates) and 37 lineage II isolates (72.5% of the 51 lineage II isolates) (S1 Table); this difference was significant (p<0.05). The shortest length of combined plasmid contigs of any isolate was 38 Kbp. Combined plasmid contigs from a CC5 isolate and a ST635 isolate were 149 Kbp and 137 Kbp, respectively. All other isolates had combined plasmid contigs between 58 Kbp and 90 Kbp. *repA* was present in plasmid contigs of these 49 isolates, further suggesting that these isolates contained plasmids. All these plasmid-carrying isolates contained either *cadA1C1* or *cadA2C2*. Two CC5 isolates (CFSAN028698 and CFSAN028794) and the ST635 isolate (CFSAN028802) had both *cadA1C1* and *cadA2C2*, and among them CFSAN028698 had both cadmium resistance cassettes in the same plasmid contig. Among the 49 plasmid-carrying isolates, 45 (91.8%) had *bcrABC*, suggesting high correlation between plasmid presence and BC tolerance in the *Lm* MBS collection. Among the 9 clones that contained at least 5 isolates per clone, all CC5, CC199, CC321, CC7 and CC9 isolates contained plasmid(s); and none of the CC87 or CC6 isolates contained plasmid(s). We then compared results of plasmid analysis between the two duplicate isolates of each positive sample. In one sample, the isolate (CFSAN028806) we included for further genetic analysis contained plasmid(s) of 72 Kbp and the other isolate (CFSAN028805) had no plasmid contigs (S1 Table). It is possible that the plasmid was lost during culturing. In each of other samples, duplicate isolates yielded the same result on the presence/absence of plasmids, and among those plasmid-carrying isolates, the lengths of plasmid contigs between duplicate isolates differed by ≤5%, indicating decent coverage of plasmids by shotgun sequencing.

This study and previous studies [23, 30, 32] determined the presence of specific genes for virulence and stress response of *L*. *monocytogenes* using WGS data. This study shows that certain genes involved in tolerance to salt and oxidative stress (e.g., SSI-1, SSI-2), tolerance to benzalkonium chloride sanitizer (e.g., *bcrABC*), and biofilm formation (e.g., *bapL*) were more likely to occur in lineage II isolates. Findings reported in other studies [19, 30, 32] suggested that *L*. *monocytogenes* adapted to food and food processing environments had a higher prevalence of genes involved in stress resistance and tolerance to benzalkonium chloride. Notably, our collection of isolates were all from food sources, while some previous studies included isolates from none-food sources. The WGS data generated herein should be useful for future studies to investigate phenotypes that could offer more insights on the virulence, stress response, and environmental persistence potential of the isolates of various genotypes found in this study.

### Identification of possible transmission and persistence events

Analysis of the WGS data identified several clusters among the isolates in our collection. The *Lm* MBS was conducted to primarily determine prevalence, levels, and types of *L*. *monocytogenes*, not specifically for identifying transmission or persistence events. The collected metadata included, among other information, food (and list of ingredients on the label), food category, food group, type of store (e.g., national chain or independent), store ID (whether the same store was visited multiple times over two years), product packaging location (prepackaged vs. deli-packaged), sampling date, and the FoodNet site [13]. Except for sprout samples, the survey was blinded with regard to the store name, the brand, and the manufacturer. Nonetheless, the available metadata were useful in the analysis of isolate clusters, which could subsequently aid in the investigation of possible cross contamination and persistence events.

Six clusters, Clusters A through F (Table 3), included isolates obtained from food samples prepackaged in manufacturing facilities and shipped to retail stores. The information about these clusters could aid in the investigation of whether different samples were produced and prepackaged by a common manufacturer and transported to the same or different stores. Cluster A had two CC7 isolates that displayed no allelic differences. One of the isolates was obtained from a broccoli sprout sample and the other from an alfalfa sprout sample; both samples were prepackaged and produced by the same manufacturer and sold in the same store in Georgia. Cluster B had two CC6 isolates with no allelic differences, which were recovered from two prepackaged soft-ripened and semisoft cheese samples in two different stores in Maryland one month apart. Cluster C included two CC88 isolates differing by 3 alleles, which were recovered from two prepackaged smoked salmon samples in a Georgia store on the same day. Cluster D included three CC379 isolates differing by ≤1 allele, which were recovered from prepackaged raw milk samples collected in two different stores in Connecticut on the same day. Clusters E&F were prepackaged raw cut vegetable samples with each cluster purchased in one Maryland store: Cluster E had two CC131 isolates differing by 1 allele and Cluster F had two ST489 isolates with no allelic differences.

**Table 3:** Clusters of isolates involved in possible transmission and persistence events.

CFSAN ID	Clone	Cluster ID	Collection date	Food	State	Packaging location
CFSAN012230	CC7	A	2014-01-06	Alfalfa sprouts	GA	Prepackaged
CFSAN012299	CC7	A	2014-01-06	Broccoli sprouts	GA	Prepackaged
CFSAN028792	CC6	B	2013-01-30	Soft-ripened cheese	MD	Prepackaged
CFSAN028804	CC6	B	2013-02-27	Semisoft cheese	MD	Prepackaged
CFSAN028694	CC88	C	2011-06-04	Cold smoked salmon	GA	Prepackaged
CFSAN028696	CC88	C	2011-06-04	Cold smoked salmon	GA	Prepackaged
CFSAN028732	CC379	D	2011-12-07	Raw milk	CT	Prepackaged
CFSAN028734	CC379	D	2011-12-07	Raw milk	CT	Prepackaged
CFSAN028736	CC379	D	2011-12-07	Raw milk	CT	Prepackaged
CFSAN028758	CC131	E	2012-09-15	Raw, cut vegetables	MD	Prepackaged
CFSAN028760	CC131	E	2012-09-15	Raw, cut vegetables	MD	Prepackaged
CFSAN028750	ST489	F	2012-07-07	Raw, cut vegetables	MD	Prepackaged
CFSAN028895	ST489	F	2012-07-07	Raw, cut vegetables	MD	Prepackaged
CFSAN028682	CC155	G	2011-04-02	Turkey salad	MD	Made-in-Store
CFSAN028684	CC155	G	2011-04-02	Chicken salad	MD	Made-in-Store
CFSAN028678	ST376	H	2011-04-02	Shrimp seafood salad	MD	Made-in-Store
CFSAN028680	ST376	H	2011-04-02	Shrimp seafood salad	MD	Made-in-Store
CFSAN028686	ST429	I	2011-04-16	Egg salad	CA	Made-in-Store
CFSAN028688	ST429	I	2011-04-16	Egg sandwich	CA	Made-in-Store
CFSAN028712	CC155	J	2011-08-04	Egg sandwich	MD	Made-in-Store
CFSAN028718	CC155	J	2011-11-02	Egg salad	MD	Made-in-Store
CFSAN028720	CC155	J	2011-11-02	Egg sandwich	MD	Made-in-Store
CFSAN028722	CC155	J	2011-05-28	Egg sandwich	MD	Made-in-Store
CFSAN028744	CC155	J	2012-05-04	Egg salad	MD	Made-in-Store
CFSAN028764	CC155	J	2012-11-24	Egg salad	MD	Made-in-Store
CFSAN028776	CC155	J	2013-01-05	Egg salad	MD	Prepackaged
CFSAN028666	CC9	K	2011-03-05	Cantaloupe, fresh-cut	GA	Made-in-Store
CFSAN028668	CC9	K	2011-03-05	Cantaloupe, fresh-cut	GA	Made-in-Store
CFSAN028670	CC9	K	2011-03-05	Cantaloupe, fresh-cut	GA	Made-in-Store
CFSAN028672	CC9	K	2011-03-05	Mixed fruit, fresh-cut	GA	Prepackaged
CFSAN028730	CC199	L	2011-12-07	Egg salad	CT	Made-in-Store
CFSAN028740	CC199	L	2012-03-30	Egg salad	CT	Made-in-Store
CFSAN028742	CC199	L	2012-03-30	Egg salad	CT	Prepackaged
CFSAN028674	CC155	M	2011-03-25	Finfish salad	CA	Prepackaged
CFSAN028676	CC155	M	2011-03-25	Mixed seafood salad	CA	Made-in-Store
CFSAN028766	CC155	N	2012-11-24	Potato salad	MD	Made-in-Store
CFSAN028768	CC155	N	2012-11-24	Potato salad	MD	Made-in-Store
CFSAN028773	CC155	N	2013-01-05	Egg salad	MD	Prepackaged
CFSAN028778	CC155	N	2013-01-05	Potato salad	MD	Made-in-Store
CFSAN028780	CC155	N	2013-01-05	Potato salad	MD	Made-in-Store

Clusters G, H, and I (Table 3) were from made-in-store samples (i.e., product was made or packaged at the deli in a retail store). Metadata for these clusters could aid in the investigation of possible cross contamination of different product types during preparation at the retail deli or help generate hypotheses on the common contamination source(s) or ingredient(s). Cluster G included two CC155 isolates with no allelic differences, which were recovered from two made-in-store samples (i.e., chicken salad and turkey salad) collected in a Maryland store on the same day. Cluster H had two ST376 isolates differing by 3 alleles, which were recovered from two shrimp salad samples in a Maryland store on the same day. Cluster I had two ST429 isolates differing by 3 alleles, which were recovered from two made-in-store samples (i.e., egg salad and egg sandwich) in a California store on the same day.

Still other clusters consisted of isolates from samples of seemingly diverse product categories and sampling locations. Cluster J (Table 3) consisted of seven CC155 isolates differing by ≤3 alleles, from three made-in-store egg salad samples, one prepackaged egg salad sample, and three made-in-store egg sandwich samples. These seven samples were purchased from seven different stores in Maryland between May 2011 and January 2013. Such information could be useful for generating hypotheses for investigating contamination source(s) due to common ingredient(s) or supplier(s) such as: whether the stores received egg salad from a central manufacturing facility and re-packaged the product for sale at the deli counter; whether the stores used the egg salad to make egg sandwiches; whether the contamination came from the processing environment and/or from common ingredient(s) in the egg salads and egg sandwiches (product label indicated that these samples contain diced vegetables in addition to eggs and other ingredients). It is not uncommon that retail stores might obtain deli-type salad in bulk from a manufacturing facility and re-package and sell them at the deli counter (which would be documented as “made-in-store” in the *Lm* MBS survey).

In each of Clusters K, L, M and N (Table 3), isolates were obtained from prepackaged and made-in-store samples. Cluster K had four CC9 isolates differing by ≤3 alleles, which were recovered from four samples in a Georgia store on the same day; three of the samples were made-in-store cut cantaloupes and one of the samples was prepackaged mixed fruit that may have contained cantaloupe. Cluster L had three CC199 isolates with no allelic differences, which were recovered from three egg salad samples in Connecticut; two samples (one prepackaged and one made-in-store) were collected in the same store on the same day and the third sample (made-in-store) was collected in a different store on a different day. Cluster M had two CC155 isolates with no allelic differences, which were recovered from two samples (i.e., prepackaged finfish salad and made-in-store mixed seafood salad that contained salmon) collected in two stores in California. Cluster N included five CC155 isolates differing by ≤3 alleles, which were recovered from five different samples in two stores of Maryland. Two made-in-store potato salad samples were collected in one store on the same day; two made-in-store potato salad samples and one prepackaged egg salad were collected in another store on the same day.

When the cgMLST scheme was used to analyze more than 40 previous listeriosis outbreak strains [25], epidemiologically-related isolates belonging to most outbreak strains differed by a maximum of 12 alleles. The maximum difference between any neighboring isolates (i.e., linkage) in a minimum-spanning tree of most outbreak strains was 9 alleles. Isolates in the clusters (Table 3) discussed above were well below the previously determined genetic diversity among epidemiologically-related isolates.

### Conclusions and future work

The collection of *L*. *monocytogenes* isolates from the 2010–2013 *Lm* MBS survey [13] offered an unique opportunity via WGS subtyping to gain insights into strain diversity, relatedness, baseline CCs, and virulence gene profiles of isolates recovered from RTE foods commonly consumed in the U.S. Prevalence of genetic lineages, serogroups and CCs were determined, with proportions of lineage I and lineage II isolates being 48.0% and 50.0%, respectively. Serogroup IIa, IIb and IVb were the most dominant serogroups, containing 93.2% of the isolates. There is no significant difference in the genetic diversity of isolates recovered from different food groups. Presence of PMSCs of *inlA*, major virulence genes and genes involved in stress response were determined. The findings offer valuable insights on the genetic differences between lineage I and lineage II isolates. The present study found significant shifts toward a higher proportion of lineage I isolates and a higher proportion of isolates with intact inlA among food isolates compared with isolates collected from RTE food groups in a similar survey in 2000–2001, which may suggest a shift toward a higher average virulence potential in *L*. *monocytogenes* in the U.S.

An implicit underlying assumption in published whole-genome-based studies [19, 29, 30, 33] is related to the spatial and temporal representativeness of the strains upon which the frequency of occurrence for a genetic subtype is determined. Results from this study again illustrate the powerful capacity of WGS to identify clusters of closely related isolates, as well as the value of well-documented metadata critical to evaluate genetic diversity of isolates from different RTE food groups in a baseline exposure situation.

This study was conducted in part to address recommendations from the Interagency Risk Assessment Consortium (IRAC) Work Group on the Application of Whole Genome Sequencing to Assess Food Safety Risk [79]. With the rapid advancements in pathogen subtyping and broad acceptance and use of WGS data by regulatory agencies for foodborne outbreak detection and source tracking [29, 33, 80, 81], a multi-disciplinary approach is needed to explore how WGS data may be used to assess and manage risks from foodborne pathogens, including *L*. *monocytogenes* [79].

A substantial benefit of WGS data for *L*. *monocytogenes* isolates from large-scale comprehensive surveys is the overall relatedness of strains with metadata for time, location, and food type. Importantly, WGS with sample metadata could inform future risk assessments. For example, these data could better define *L*. *monocytogenes* subgroups (such as lineages and clones), estimate the prevalence and levels of genetic subtypes in food, identify strains with well-defined genetic profiles for use in growth and survival studies of both foods and environments.

The work described here is limited to the 201 isolates retained from the 2010–2013 *Lm* MBS survey. Future work is needed to compare the WGS dataset from this study with WGS datasets from clinical isolates from the same timeframe and geographic regions, to identify clusters and determine potential linkages to human listeriosis cases and outbreaks, taking into consideration temporal, microbiological, and epidemiological evidence. Identifying genotypes that are significantly associated with clinical cases and those that are not could facilitate the evaluation of virulence potential of different genotypes of *L*. *monocytogenes*. This information can be critical in assessing the risk of *L*. *monocytogenes* infection in a population subgroup or a single patient. NCBI Pathogen Detection tool (https://www.ncbi.nlm.nih.gov/pathogens) clusters and identifies related genomes that are deposited into GenomeTrakr, which could be used to identify clinical isolates related to the isolate in the 2010–2013 *Lm* MBS survey. In addition, such clusters and curated metadata can be integrated in GenomeGraphR, a web-based visualization and analytic tool, to investigate routes of transmission, to identify persistence and virulence phenotypes, to assess exposure and to characterize hazard [82]. Indeed, WGS data from food and clinical isolates, together with metadata, could also be used to refine assumptions underlying an existing dose-response model [10]. For example, WGS data could be used to further refine *L*. *monocytogenes* dose-response relationships with adjustments for variability in strain virulence and host susceptibility. Future studies could be designed to guide the collection of WGS data and related metadata to further leverage the potential impact of WGS technology with food safety risk assessment methodology to enhance the types of risk management decisions it can inform.

## Supporting information

S1 Fig (PDF)

S2 Fig (PDF)

S1 Table List of published complete sequences of Listeria plasmids available at NCBI. (XLS)

S2 Table Results for 195 L. monocytogenes isolates from the 2010–2013 LmMBS^a^. (XLSX)
