# Supplementary figures and images for: Genetic diversity and profiles of genes associated with virulence and stress resistance among isolates from the 2010-2013 interagency Listeria monocytogenes market basket survey
Source: PLoS One. 2020 Apr 30;15(4):e0231393. doi: 10.1371/journal.pone.0231393 (PMC7192433; doi:10.1371/journal.pone.0231393)

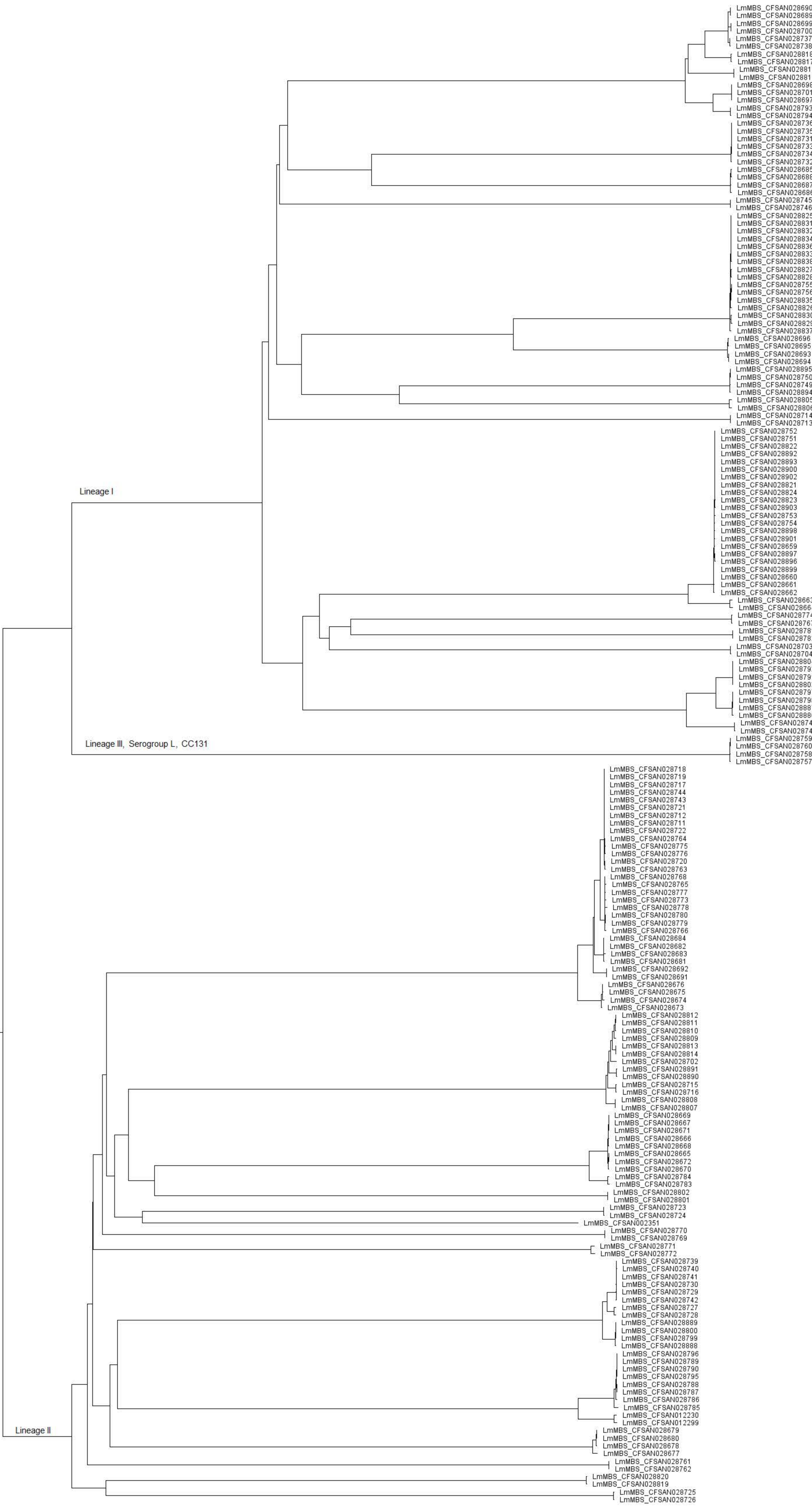

Supplement: S1 Fig — (PDF) [file pone.0231393.s001.pdf]
